# A Mobile Phone App for the Generation and Characterization of Motor Habits

**DOI:** 10.3389/fpsyg.2019.02850

**Published:** 2020-01-08

**Authors:** Paula Banca, Daniel McNamee, Thomas Piercy, Qiang Luo, Trevor W. Robbins

**Affiliations:** ^1^Department of Psychology, Behavioural and Clinical Neuroscience Institute, University of Cambridge, Cambridge, United Kingdom; ^2^Wellcome Centre for Human Neuroimaging, Institute of Neurology, University College London, London, United Kingdom; ^3^Max Planck UCL Centre for Computational Psychiatry, University College London, London, United Kingdom; ^4^Department of Psychiatry, Addenbrooke’s Hospital, University of Cambridge, Cambridge, United Kingdom; ^5^Institute of Science and Technology for Brain-Inspired Intelligence, Fudan University, Shanghai, China; ^6^Key Laboratory of Computational Neuroscience and Brain-Inspired Intelligence, Ministry of Education, Fudan University, Shanghai, China; ^7^State Key Laboratory of Medical Neurobiology, MOE Frontiers Center for Brain Science, Institute of Brain Science and Human Phenome Institute, Fudan University, Shanghai, China

**Keywords:** habit, skill, automaticity, motor sequence learning, extinction, sequence completion times, preparation time, routine

## Abstract

Habits are a powerful route to efficiency; the ability to constantly shift between goal-directed and habitual strategies, as well as integrate them into behavioral output, is key to optimal performance in everyday life. When such ability is impaired, it may lead to loss of control and to compulsive behavior. Habits have successfully been induced and investigated in rats using methods such as overtraining stimulus-response associations and outcome devaluation, respectively. However, such methods have ineffectively measured habits in humans because (1) human habits usually involve more complex sequences of actions than in rats and (2) of pragmatic impediments posed by the extensive time (weeks or even months), it may take for routine habits to develop. We present here a novel behavioral paradigm—a mobile-phone app methodology—for inducing and measuring habits in humans during their everyday schedule and environment. It assumes that practice is key to achieve automaticity and proficiency and that the use of a hierarchical sequence of actions is the best strategy for capturing the cognitive mechanisms involved in habit formation (including “chunking”) and consolidation. The task is a gamified self-instructed and self-paced app on a mobile phone that enables subjects to learn and practice two sequences of finger movements, composed of chords and single presses. It involves a step-wise learning procedure in which subjects begin responding to a visual and auditory cued sequence by generating responses on the screen using four fingers. Such cues progressively disappear throughout 1 month of training, enabling the subject ultimately to master the motor skill involved. We present preliminary data for the acquisition of motor sequence learning in 29 healthy individuals, each trained over a month period. We demonstrate an asymptotic improvement in performance, as well as its automatic nature. We also report how people integrate the task into their daily routine, the development of motor precision throughout training, and the effect of intermittent reinforcement and reward extinction in habit preservation. The findings help to validate this “real world” app for measuring human habits.

## Introduction

The concept of habit learning has been extensively studied across distinct fields of research, using different methodologies (for a comprehensive review on habits, see Wood and Rünger, 2016). Habits are usually defined as automatic responses elicited by specific environmental stimuli (including contexts) performed autonomously of the goal (e.g., Lin et al., 2016; Robbins and Costa, 2017). Habits have been assumed to require practice or repeated training as demonstrated in experimental animals (e.g., Adams and Dickinson, 1981) and humans (Tricomi et al., 2009). However, it has proven to be surprisingly difficult to demonstrate robust habit learning in humans as a function of training (de Wit et al., 2018), possibly for reasons related to the time allowed for response preparation prior to execution (Hardwick et al., 2019), the need for much longer periods of training for humans than are possible in the laboratory and a focus on single actions rather than more complex sequences of behavior. Dezfouli and Balleine (2012) have argued that “habits are complex actions that reflect the association of a number of actions into rapidly executed action sequences.” Such action sequences have been understudied, especially given the evidence for the “chunking” together of elements of response sequences and their dependence on the striatum, a brain structure also associated with habit learning and performance (Graybiel, 1998; Sakai et al., 2003). Action sequences may also provide proprioceptive and kinesthetic sensory feedback that facilitates habit learning *via* the stimulus-response associations occurring as a consequence of the response chain and distal to the goal occurring at the end of the sequence. In this research, we aimed to develop a method for investigating habitual control of motor response sequences in the real world using a very familiar apparatus (the smartphone) over protracted training periods in human participants—and we report here a preliminary study aimed at validating a gamified application for this purpose.

Previously, self-reported questionnaires have been used to investigate aspects of habits distinguishing between routine and automatic tendencies in humans (Gardner et al., 2012; Ersche et al., 2017). These are useful but do depend on self-report rather than providing more objective measures of habits.

“Ecological” paradigms have also been used to track “real-world” habits (Lally et al., 2010; Fournier et al., 2017) assessing, among other elements, the “four horsemen of automaticity” as defined by Bargh and colleagues: awareness, intention, efficiency, and control (Bargh, 1994). Hence, we incorporated measures of automaticity of our response sequences, including speed, accuracy, and motor invariance.

Capitalizing on novel technology, we developed a smartphone motor sequence application to measure habit formation within a more naturalistic setting (at home). Habit strength is promoted here by the permanent accessibility of the app (given that most people carry their mobile phones everywhere), which facilitates training frequency and enables context stability since the tactile, visual, and auditory stimuli associated with the phone and its operation establishes a strong context for all participants regardless of their concurrent circumstances. Thus phone-based tasks favor habit formation since as the frequency of the behavior increases in a stable context, so it increases the strength of the context-behavior association, an effect that is crucial for habit development (Verplanken and Wood, 2006). Indeed, mobile phones are notorious for their elicitation of absent-minded and unintentional use patterns which are suggested to be characteristic of automated behaviors (Bargh, 1994).

We continuously collected data online, in real time, thus enabling measures of progressive learning and of processes involved in habit formation such as “caching” (Haith and Krakauer, 2018) and “chunking” (Graybiel, 1998). Previous studies have shown that practice in itself is insufficient for habit development as it requires off-line consolidation computations, through longer periods of time (de Wit et al., 2018) and sleep (Walker et al., 2003; Nusbaum et al., 2018). This article presents the method in detail and preliminary data, acquired with 29 healthy human volunteers. Specifically, we report data on task engagement and how people integrated the task into their daily routine. We also report objective accuracy data and sequence completion times throughout a 30-day training period in order to measure task-related automaticity and motor precision.

The application incorporated attractive sensory features in a game-like setting, in which participants earned reward points according to their performance (see video for illustration in the “Methods” section). This app-based method for measuring habits in the real world is based on previous findings that have defined training frequency, context stability, and reward contingencies as important for increasing habit strength (Verplanken and Wood, 2006; Wood and Rünger, 2016). Previous work in experimental animals (Dickinson et al., 1983) has shown that the schedule of reinforcement (or reward) employed affects the speed of habit learning. Hence, we employed both continuous reinforcement (where each correct sequence received reward) and a more probabilistic schedule of rewards for correct sequences, with the hypothesis that the weaker correlation of correct sequences with reward would weaken goal-directed behavior in favor of habitual learning.

In order to assess the autonomy of habits from goal-directed actions behavioral neuroscientists employ goal devaluation or contingency degradation strategies as interventions to probe habitual control (Dickinson and Weiskrantz, 1985; Tricomi et al., 2009). Although such interventions may unmask habits only indirectly by removing goal-directed control (Gillan et al., 2015; Robbins and Costa, 2017) both rodent and human studies using them have successfully shown that well-learned action sequences can indeed become habitual and are hierarchically organized such that distinct decision-making processes may differentially control the initiation and execution of sequences (Dezfouli and Balleine, 2013; Garr and Delamater, 2019). We further report here the outcome of extinction (a form of contingency degradation; Balleine and Dickinson, 1998), by removing all the reward feedback stimuli and therefore determining how performance of the sequence was maintained.

## Materials and Methods

### Participants

Twenty-nine volunteers, recruited from the community *via* advertisements (flyers), participated in the present study (11 males/18 females, mean age: 39.14 ± 11.79 years). They were all in good health, unmedicated, had no history of neurological or psychiatric conditions, and were also free from any substance dependence. Two participants who scored above 4 on the Beck Depression Scale (Beck et al., 1961) and higher than 6 on the Montgomery-Åsberg Depression Rating Scale (Montgomery and Asberg, 1979) were excluded. Only one of our recruited participants used to play video games. All participants were given a letter of information, gave written informed consent prior to participation, in accordance with the Declaration of Helsinki, and were financially compensated for their participation (£20 in total: £5 incentive each week for keeping their motivation). They were told that this research aims at investigating how habits are formed, and therefore, we would need them to repeat the task for a longer period (1 month) than in usual studies. This study was approved by the East of England–Cambridge South Research Ethics Committee (16/EE/0465).

### Habit Training Task Design

**Figure 1:**
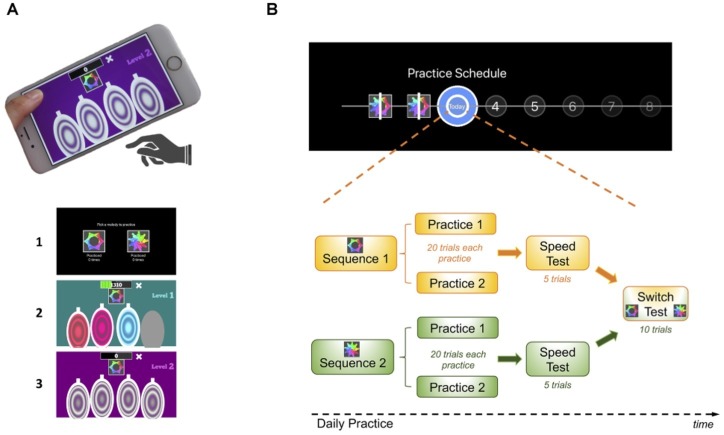
Habit Training Task. **(A)** App setup and screenshot examples of the task design: (1) sequence selection panel, each sequence identified by an abstract picture; (2) panel exemplifying difficulty level 1; (3) panel exemplifying difficulty level 2. **(B)** Description of the daily practice schedule comprising its components: practice, speed and switch tests. Each day subjects performed both sequences in a self-determined order. After a 20-trial practice of each sequence, subjects received a speed test where they were instructed to perform the sequence as fast as possible. In a final phase (after concluding the practice of both sequences), they were given a switch test, where they were cued by the sequence-associated pictures to switch between the two practiced sequences in a pseudo-random order.

The task consisted of a motor practice program that participants committed to pursue daily, for a period of 1 month (see description of the task design in Figure 1 and in the following video: https://youtu.be/XSYrBzD7ZpI).

Using a simple and self-instructed application downloaded to their mobile devices, participants learned and practiced two sequences of fingers movements, composed of chords (two or three simultaneous finger presses) and single presses (one finger only). Each sequence comprised six moves, performed using four fingers of the dominant hand (index, middle, ring, and little finger). Sequence generation was randomized so that each participant had their own pair of sequences to practice throughout the month. This randomization was conducted to rule out finger-specific effects at individual sequential positions, as each finger will contribute equally to the RTs at each sequential position. However, for each sequence, the order of finger movements was pseudo-randomly generated such that (1) all sequences had three single press moves, two two-finger chord moves, and one three-finger chord move and (2) difficult finger combinations were avoided, for example, a three-finger chord with simultaneous index, middle, and little fingers or index, ring, and little fingers. Therefore, despite being different, all sequences had a similar level of difficulty.

Participants were instructed to respond swiftly and accurately. They were required to keep their fingers very close to the keys to minimize amplitude variation and to enable them to play quickly. To enable sequence learning and memorization, three levels of increased difficulty guided practice. Initially, subjects responded to a visually and auditory cued sequence: they simply followed lighted keys, also associated with musical notes (level 1). These exteroceptive cues were slowly removed throughout the practice progression such that level 2 only included auditory cues and level 3 contained no cues. Successful performance at each stage resulted in progression to the next. Unsuccessful performance resulted in titration to the immediately preceding stage.

Participants received continuous feedback on their performance. Successful trials were followed by a positive ring tone and mistakes by a negative ring tone. Every time a mistake occurred (irrespective of which move in the sequence they were), participants had to restart the sequence in order to perform it entirely correctly.

As previously mentioned, all participants had to practice two motor sequences, each identified by a specific abstract picture. Each sequence was associated with a specific reward schedule. In our design, one of the reward schedules was continuous reward (points were received for every successful trial, as a function of the speed of performance) and the other a variable reward schedule (points were randomly received on 37% of the trials). Calculation of the points was as follows: points decreased linearly from 100 to 0 over 1 second; the counting started as soon as the app became ready to receive the user’s input. This counter reset and restarted counting after each move. As soon as the keys were pressed (for each move), the counter stopped and registered the points achieved for that move. The points received after each sequence was completed were the sum of all the points achieved on each move. All this within-move counting was done in the background so participants only saw the points gained for each sequence once they completed it. This system was implemented to promote speed: the faster participants played the sequence, the more points they gained. If they were too slow, that is, if the key press occurred after the counter had reached 0, then no points were gathered for that particular move. In the continuous reward sequence, participants received the total points acquired after each successful trial completed. In the variable reward sequence, there was a 63% chance that any points earned on a sequence would be set to zero. To compensate for the missing points, the earned points provided on this schedule were doubled. Therefore, both sequences resulted in similar scores by the end of practice. By this point, after 20 sequences had been completed (see “Practice Schedule” section below), subjects could see the total (cumulative) points achieved throughout the practice. While playing, they could also see their current total, gathered at a particular moment. To promote motivation, feedback was also given across daily practice sessions, so subjects could compare their performance across practices and see whether they were improving over days.

### Practice Schedule

All participants were presented with a calendar schedule and were asked to practice both sequences daily (Figure 1B). They were instructed to practice as many times as they wish, whenever they wanted during the day and with the sequence order they would prefer. However, a minimum of two practice sessions per sequence was required every day; each practice comprised 20 sequences. The instruction was the following: “*You can practice as many times as you wish, whenever you want during your day and with the sequence order you want. Your minimum training required per day is 2 rounds of practice for each sequence but since every person has different learning rates, you are responsible for assessing how much you need to practice in order to make sure you come back for a second session, in a month time, mastering the sequences. You need to know them by heart, automatically and quickly!*”. Once the minimum practice sessions were completed, a short retention speed test of five trials followed, to assess that day’s performance. During this short session, participants were instructed to repeatedly tap a sequence as rapidly as possible while making as few errors as possible. After this, participants were asked to rate, on a percentage scale, the following two questions: (1) *How much did you enjoy playing this sequence?* and (2) *How confident are you that you know this sequence by heart?* Finally, participants were required to engage in a 10 trial-switch test, in which they would practice switching between the two sequences in a pseudo-random order. The sequence to be played was cued by the respective associated picture. Speed and switch tests never received reward feedback (only the practice sessions). This sequence of events (practice, speed, ratings and switch sessions) happened every day (Figure 1B). If subjects would miss a day of practice, they would need to catch up on the training the day after, that is, they would be required to do the minimum training for the current and previous day. To remove pauses in the training, a “dead man” switch procedure was implemented in the app.

Thirty days of practice were required, and all data were anonymously collected in real time, through an online server. At the 21st day of practice, the reward schedules were removed (extinction) to test how autonomous of external feedback the response sequence had become. This procedure (1) ensured that the response sequence was more dependent on interoceptive (proprioceptive and kinesthetic) feedback and on the subjects’ internal motivation to continue the training and (2) ensured that we were able to measure and train response sequences triggered by their context, which persisted without explicit reinforcement.

An orientation session, lasting between 30 and 60 min depending on people’s dexterity, was conducted at the Herschel Smith Building, Addenbrooke’s Hospital, in Cambridge. During this session, the researcher helped the participant to download the app to their devices, reviewed the training instructions, and discussed how the task works. All participants were instructed to practice every day to make sure they could perform both sequences automatically and rapidly as they would be assessed in a second session taking place 1 month later. This cover story was introduced in preparation for a follow-up session including a devaluation strategy, which assessed participants’ preferences for habitual sequences over goal-seeking sequences. This task manipulation would test the hypothesis that the behavioral mechanism underlying the transition from a goal-directed to a habitual action is that the action, with repetition, acquires the rewarding properties of its outcome, which may simply be its own proprioceptive/kinesthetic feedback (data to be reported elsewhere).

### Data Analyses

Behavioral output measures included sequence accuracy and sequence completion times (learning rates), temporal pattern of daily practice, days until habit acquisition, performance as function of different reward schedules, effect of reward extinction and finger position and timings.

For more detailed analyses, we broke down the sequence completion times into two components: (1) *move preparation time*: the time period between the last release of the previous move and the first press of the current move and (2) *move performance time*: the time period between the first press and the last release of each move, representing the duration of each move from the time participants press until they release the keys (i.e., muscle time).

App data were automatically uploaded to a Cloud-based database. Data analysis was performed using custom scripts in MATLAB and Python.

## Results

### Validation of Training and Individual Routines

**Figure 2:**
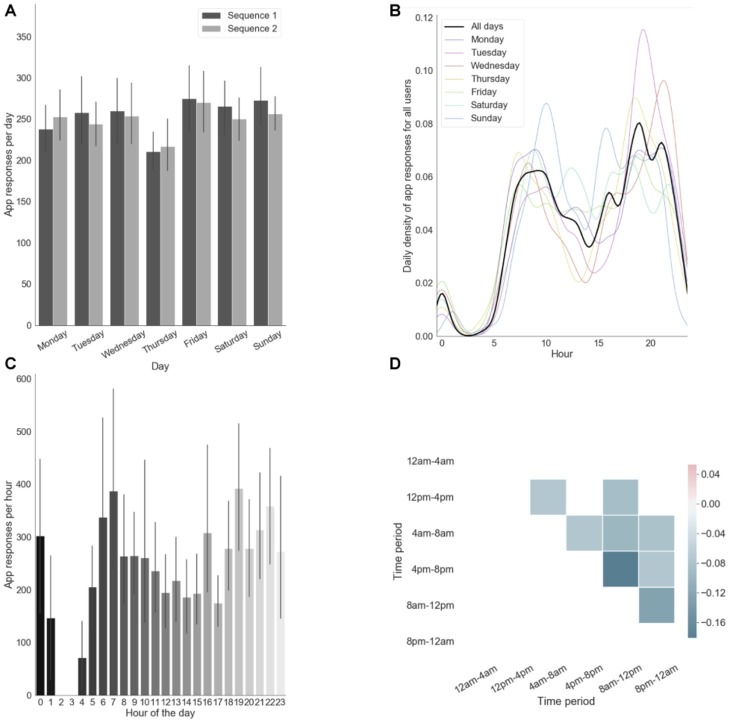
App engagement. **(A)** App responses (i.e., number of touches) per day computed separately for each motor sequence. **(B)** Probability of app responses per hour for each days. **(C)** App responses per hour in the day. Error bars reflect standard error of the mean across subjects. **(D)** Correlation matrix of app engagements per daily time period (only significant correlations are shown, *p* < 0.05).

As shown in Figures 2A–D, our participants reliably committed to their regular training schedule. They generally fulfilled the requirement of practicing consistently both sequences every day (Figure 2A). The approximately bimodal distribution observed in Figure 2C depicts our participant’s tendency to practice mostly during early mornings (~7:00) and evenings (~19:00). This tendency was relatively consistent across days (Figure 1B). Moreover, on a daily basis, participants typically chose to practice at one time point of their day as shown by the anti-correlations in app engagement across different daily time periods (Figure 2D). In particular, those who chose to practice in the evening tended not do it in the morning and vice versa, as indicated by the strongest anti-correlation between 8 and 12 am and 4 and 8 pm.

### Effects of Extinction

**Figure 3:**
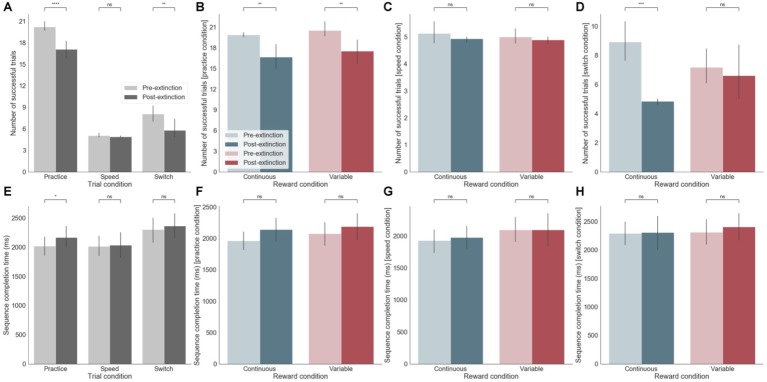
**(A-D)** Effect of extinction on number of successful trials. **(A)** Across the three different training sessions (practice, speed, and switch) pre- and post-extinction. **(B)** Separately for continuous and variable reward conditions for practice sessions only. **(C)** Separately for continuous and variable reward conditions for speed sessions only. **(D)** Separately for continuous and variable reward conditions for switch sessions only. **(E-H)** same as **(A-D)** but for sequence completion time. ns: *p* > 0.05; *: 0.01 < *p* <= 0.05; **: 0.001 < *p* <= 0.01; ***: 0.0001 < *p* <= 0.001; ****: *p* <= 0.0001.

We analyzed the two blocks of practice pre- and post-removal of the external rewarding feedback occurring after 21 days of training. After *extinction*, during the practice session, there was a significant decrease in performance in terms of both increased errors (*p* < 0.0001) and longer sequence completion times (*p* < 0.05) (Figures 3A,E). This effect occurred irrespective of the reward feedback schedule (continuous versus variable, Figure 3B). Nevertheless, analyses of subsequent effects of *extinction* on the switch and speed tests (although these had never previously received reward feedback) showed that there was a significant performance decrement post-*extinction* during the switch test, only following continuous reward feedback training (*p* < 0.001) (Figure 3D). There was however no effect on sequence completion time (Figure 3H). There was no effect on post-extinction performance during the speed test (Figures 3C,G). In summary, participants made significantly more errors after *extinction* in both sequences, irrespective of whether successful sequences were previously rewarding in a continuous or variable manner. During the switch test, this accuracy effect was only strongly observed for the continuous reward. Generally, accuracy seemed to be strongly affected by reward *extinction* (Figure 3, top row - number of successful trials) but sequence completion times were less sensitive to this manipulation (Figure 3, bottom row - sequence completion times).

### Sequence Performance

**Figure 4:**
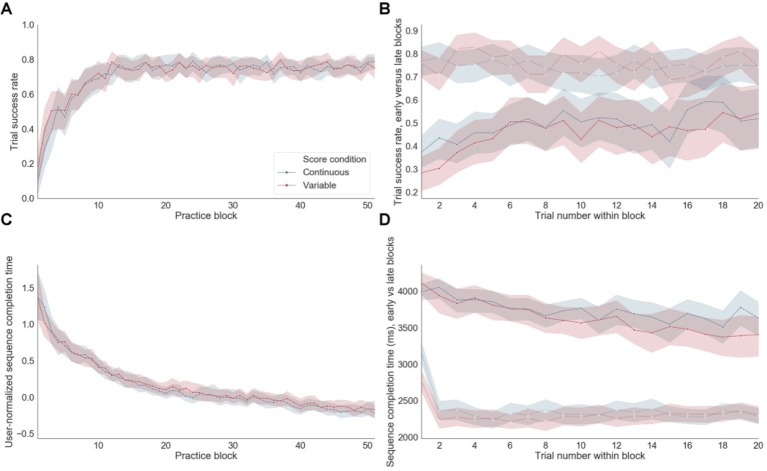
Performance. **(A)** Trial success rate (i.e., rate of correct sequences) over practice training. **(B)** Trial success rate in early versus late blocks of practice. Dashed line: late blocks, Continuous line: early blocks. **(C)** Sequence completion time, normalized within each subject, on successful trials over practice. **(D)** Sequence completion time in successful trials in early versus late blocks of practice. Dashed line: late blocks, Continuous line: early blocks.

Significant improvements in accuracy (Figure 4A) and normalized group-averaged decreases in sequence completion times (Figure 4C) throughout training indicate that learning occurred as expected. Participants started their training with a mean sequence completion of 3,719 ms in successful trials based on the first five blocks of practice (referred to as “early training”) and completed their training with a mean sequence completion time of 2,346 ms calculated using the last five blocks of practice (late training). A paired *t*-test between the mean sequence completion time per subject in the early versus late training periods was significant at *p* < 10^−20^ (Figure 4D). Accuracy also improved significantly (p < 10^−^7) from early (mean success rate = 0.46) versus late (mean success rate = 0.75) training, with steep improvements occurring at the beginning of training and remaining stable to the end of app engagement (Figure 4B). There were no significant differences for either errors or sequence completion times as a function of the reward feedback schedule (i.e., continuous versus variable). For errors, performance appeared to reach an asymptote between blocks 15 and 20. In contrast, for sequence completion time, performance continued to improve throughout training suggesting that these behavioral measures are differentially sensitive to distinct learning processes. Throughout training, the sequence completion time of the first trial within each block appeared to be longer than subsequent trials within the same block (Figure 4D, dashed lines).

**Figure 5:**
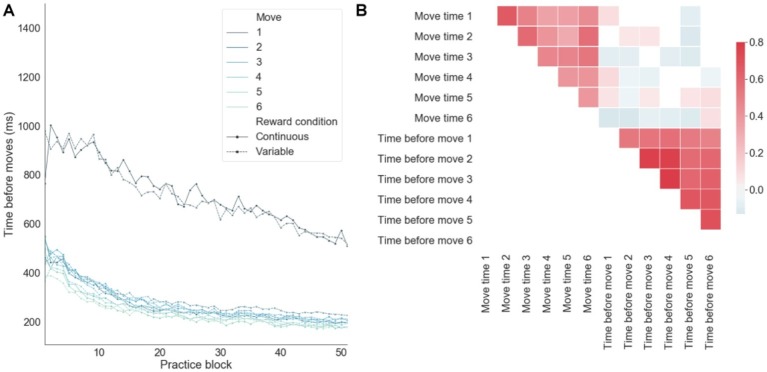
Decomposing sequence completion times into preparation and motor related components. **(A)** Preparation time (time before moves) as a function of practice for each move in each sequence averaged across subjects. **(B)** Correlations between motor (move time) and preparation times.

When decomposing the sequence completion time on successful trials into preparation (i.e., quantifying the time just before a move) and motor-related components (Figure 5A), there was an order effect by which the move number inversely correlated with the preparation time, consistent with a competitive queuing model of action sequence preparation (Averbeck et al., 2002; Rhodes et al., 2004). That is, as the sequence is performed successfully, fewer moves compete for motor output, thus resulting in shorter preparation times. There was a significantly larger preparation time for the first move, as compared with all the remaining moves of the sequence (Figure 5A). This time period before the first move also includes the time devoted to the sensory processing of the input stimuli from the app. The linear decrease in sensorimotor processing before the first move over training was in contrast to the exponential decay toward baseline observed in the remaining moves. This suggests that qualitatively different learning processes are engaged by the brain in order to optimize sensory-to-motor and motor-to-motor mappings. In correlation analyses (Figure 5B), it was found that move preparation times and move motor times were (separately) strongly correlated, whereas preparation times and motor times were weakly anti-correlated. This suggests that, over learning, preparation times and motor times were improved in a consistent manner across moves and that the brain may trade-off preparation and motor times in order to achieve an efficient balance between speed and accuracy. In particular, the anti-correlation between preparation and motor times on successful trials emerged due to trials with both fast preparation and fast motor times leading to errors. In summary, despite theoretical and empirical dependencies between these two components of the RT, there was some degree of independence between them as reflected in the relatively lower correlation cross-component correlation values.

### Motor Precision

**Figure 6:**
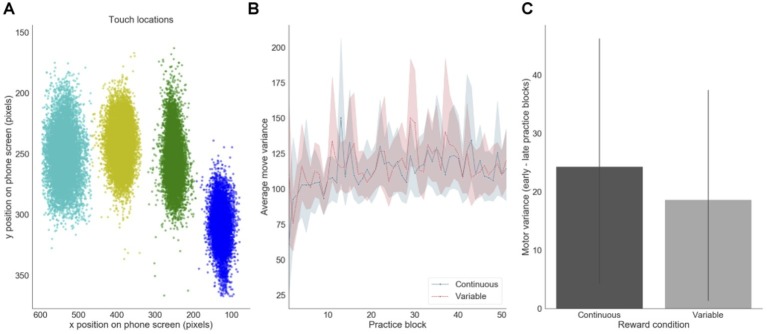
Motor precision. **(A)** Distribution of touch locations on the screen (example of one subject). **(B)** Finger precision variance over practice training. **(C)** Difference in finger variance between early and late training for each reward feedback schedule (presented for the continuous and variable sequences separately).

We also assessed how motor precision, as measured by finger position variance, varied throughout training. This measure was computed using the X and Y pixel coordinates of participants’ screen touches (Figure 6). There was a decrease in average motor precision throughout training, mainly during the first 10 blocks of practice (Figure 6B). This decrease was slightly, but not significantly, more pronounced in the continuous reward condition (*p* = 0.040) than in the variable reward one (*p* = 0.061) (Figure 6C).

## Discussion

We have presented an experimental paradigm based on motor sequence learning which can be employed to study, in a systematic and controlled way, the building blocks of more complex behavioral sequences that make up our everyday real-world actions. Designed as a smartphone tool, and thus easily available to subjects, it enabled for the first time, the induction and measurement of habitual behavior in humans during their everyday schedule, routines, and environment (in the comfort of their homes), while collecting continuously 30 days of real-time data. Such a naturalistic experimental set-up may perhaps be useful for the future investigation of habits.

The test paradigm is assumed to encompass multiple and continuous cycles of model-free and model-based learning processes thought to be required for habit development, which include processes of instrumental or operant reinforcement, adaptation, plasticity, and other explicit cognitive processes (Krakauer and Mazzoni, 2011; Haith and Krakauer, 2013). It also assumes that practice is key to achieve automaticity and proficiency and that the use of a hierarchical sequence of actions is the best strategy for capturing the cognitive mechanisms involved in habit formation and consolidation.

This app-based method to measure habits in the real world is based on previous literature which has isolated frequency, context stability, rewards, and simplicity as important factors that promote habit strength (Verplanken and Wood, 2006; Wood and Rünger, 2016). Participants perform the task on a frequent basis in a similar context (i.e., the phone and app), supported by game-related rewards. Our purpose here is to present the method in detail and validate it based on data in healthy volunteers. Our preliminary analyses attest to its successful design and good tolerability. All subjects completed the training. After 1 month of training, their speed and accuracy greatly improved. They were also capable of learning the task and performed it with a pronounced degree of automaticity. Participants reported that the task became simpler and easier to perform throughout the training, corroborating the assumption that perceived complexity of a behavior is also an element that influences the extent to which automaticity is attained (McCloskey and Johnson, 2019). In agreement with recent questionnaire methods for parsing components of habits (e.g., Ersche et al., 2017), we observed both routine (evidenced by the anti-correlation in app engagement across different daily time periods) and automaticity (evidenced by a combination of an asymptotic performance and responsiveness in the absence of cues).

Automaticity was measured in terms of three criteria: sequence completion times, progressive extinction of learning cues, and autonomy from the goal as assessed by extinction. Additionally, proficiency was also measured in terms of motor precision. The significant increase in finger variance throughout the training is also a strong indicator of motor performance optimization. According to optimal feedback control theory (Todorov and Jordan, 2002), optimal performance is achieved by allowing variability in redundant (task-irrelevant) dimensions. While still learning, participants tend to be more precise, “freezing” the degrees of freedom of their movements and having a fine-tuned and highly accurate sequence of movements (Bernstein, 1967; Vereijken et al., 1992). With training, as the skill develops into a fluid level of proficiency, motor variance increases because subjects learn that this will not impact successful sequence completion and contributes to an improved speed-accuracy trade-off (Todorov and Jordan, 2002).

In terms of proficiency and automaticity, sequence completion times significantly improved throughout training, reaching asymptotic performance levels between practice blocks 40 and 50. The exponential decay in error rates to an asymptote and further optimization of the speed/accuracy trade-off is clear evidence of learning and skill development. The greater improvement in sequence completion time during the initial 20 blocks corresponds to the “fast learning” mode, typically observed during the goal-based acquisition phase mediated by the associative striatal regions, in coordination with cerebellum, prefrontal, and premotor cortical regions (Hikosaka et al., 2002; Hardwick et al., 2013). The progressive stabilization of the sequence completion times during the remaining blocks of training likely resembles a shift to an autonomous stage of habit development (Hikosaka et al., 1999), hypothetically linked to a devolution of control to sensorimotor striatal regions (Hikosaka et al., 1999; Lehericy et al., 2005), and progressive disengagement of cognitive control hubs in the frontal and cingulate cortices (Bassett et al., 2015). The asymptotic performance attained with our task indicates that proficiency was attained as one criterion of response sequence development. Of special note also is the significantly longer sequence completion time of the first trial compared with subsequent trials within each block that occurred only during the later stages of training when asymptotic performance was observed. This may reflect the initial retrieval of the memory of the motor program into working memory and its subsequent priming on succeeding trials. This cognitive mechanism may be an initial step underlying the “chunking” process, by which elements of the motor sequence are most efficiently ordered into a motor program, well known in motor learning research (Graybiel, 1998; Sakai et al., 2003).

The preservation of this skilled behavior after extinction of the external cues, maintaining the same high level speed-accuracy trade-off, is an additional sign of automaticity and habitual control. Our findings are consistent with Hardwick et al. (2019), who also demonstrated that practice influences habits by modulating the likelihood of habit expression *via* reducing the average time of movement initiation (Hardwick et al., 2019). We also found that in later stages of the training, our participants’ response preparation times were extremely brief and unlikely to enable expression of goal-directed responses.

One test of habitual control effected in this task was extinction, involving the omission of explicit reward feedback. The removal of rewarding feedback on the 21st day of training mainly affected errors. Although there was a small effect on sequence completion time (only in the practice condition), this was much less significant, possibly indicating that performance had indeed attained a degree of autonomy from the goal. This suggests that the motor sequence had become habitual in part but still retained some sensitivity to goal despite extinction (and hence goal-directed control). Of course, this extinction manipulation did not remove all forms of motivation from performance because of the degree of intrinsic motivation that humans exhibit in such research studies.

Although one could expect different learning patterns as consequence of different reward schedules, we did not observe significant effects of the reward feedback schedule (i.e., continuous versus variable) on habit development. There was, however, a selective effect of reward schedule in performance during the switch test. The detrimental effect of extinction on this switch test depended on the previous schedule of reward feedback, specifically occurring in the continuous condition only. A possible explanation for this might be that pitting two habits against one another in an explicit choice situation recruits executive processing, hence re-engaging the goal-directed system, which may be more vulnerable to extinction in the continuously rewarded condition because the change in reward contingency is more immediate and explicit than for the variable schedule. Future studies may seek to vary the nature of intermittency of the reward schedule by explicitly comparing random ratio versus random interval schedules, the latter being associated with greater habitual control (Dickinson et al., 1983), although making such a comparison is challenging for response sequences as distinct from single actions.

This study has a few limitations and challenges to consider. Its ecological nature, enabling people to conduct the task in the comfort of their homes, including it in their everyday schedule, routines, and environment and at their own pace, partly solves the major problem of the artificial nature of previous studies. However, this feature limits the study to its behavioral nature, making it more difficult if one wants to investigate the neural basis of habit and skill development using functional imaging. There were also some technical difficulties to deliver the app on android phones, confining our recruitment to Apple users, which obviously decreased our recruitment pool of subjects. Several iPods were purchased for lending to participants in order to facilitate recruitment. Additionally, the study required careful monitoring by the researchers on a daily basis, to track participants’ commitment, gauge motivation, and send reminders when needed. Conducting this study with clinical populations might be challenging, given that some patients may not be so motivated as healthy volunteers. However, this concern has not in fact been the case with patients with OCD we have also begun to recruit, following the same procedures. Despite all the technical challenges, which also included a continuous update of the online server for data collection, such an advanced methodology was worth pursuing since it facilitated the acquisition of a large dataset, without requiring much effort from our participants.

In conclusion, this article aimed to validate a novel behavioral method for measuring motor response sequence habits in the real world using a mobile phone app. The analysis provided here is preliminary but sufficient to show that proficiency and automaticity is attained according to several different criteria. When tested in clinical populations, the method may provide new insights into the mechanisms underlying abnormal habit learning and corticostriatal functioning in psychiatric disorders and their putative contributions to compulsive behavior. Ongoing research is using this novel app to investigate the neural mechanisms of compulsive behavior in patients with OCD. More generally, this app-based approach could be deployed in a wide variety of discrete sequential production paradigms including dexterity, music, and memory training. It could also be used in the studies of individual differences, for example, to investigate whether aspects of the Big 5 predict how quickly habit/skill is developed.

## Data Availability Statement

The raw data supporting the conclusions of this article will be made available by the authors, without undue reservation, to any qualified researcher.

## Ethics Statement

All participants were given a letter of information, gave written informed consent prior to participation, in accordance with the Declaration of Helsinki, and were financially compensated for their participation. This study was approved by the Cambridge South Research Ethics Committee.

## Author Contributions

PB and TR conceived the idea and designed the method. PB and TP developed the app. PB carried out the experiment. PB and DM conducted the data analyses. QL helped with the data preparation. PB, DM, and TR wrote the manuscript.

### Conflict of Interest

The authors declare that the research was conducted in the absence of any commercial or financial relationships that could be construed as a potential conflict of interest.
